# Unraveling the complex web: pathogenesis and prevention of gestational diabetes mellitus-related fetal overgrowth

**DOI:** 10.3389/fcell.2026.1744305

**Published:** 2026-02-03

**Authors:** Xin-Yue Jiang, Deng-Wang Chen, Tao Duan, Ji-dong Zhang, Yan-Ping Ren, Jun Tan

**Affiliations:** 1 Department of Histology and Embryology, Zunyi Medical University, Zunyi, Guizhou, China; 2 Department of Immunology, Zunyi Medical University, Zunyi, Guizhou, China

**Keywords:** fetal overgrowth, fetal vasculature, gestational diabetes mellitus, insulin resistance, macrosomia

## Abstract

Fetal overgrowth, manifesting as large for gestational age or macrosomia, remains a common complication of gestational diabetes mellitus (GDM) with neonatal and long-term metabolic implications. While maternal hyperglycemia is a key driver, evidence describes the role of dysregulated placental nutrient transport involving glucose, amino acids, and lipids mediated by signaling hubs like mTOR, IGF, and AMPK. Beyond traditional metabolic axes, this review explores emerging contributors, including gut microbiota dysbiosis and extracellular vesicle mediated communication, which modulate the environment. We synthesize evidence on fetal vascular adaptations and epigenetic programming underpinning accelerated growth. Clinically, achieving euglycemia often fails to eliminate residual overgrowth risks completely. Management is evolving to integrate advanced ultrasonic markers, such as fetal abdominal fat layer thickness, and pharmacotherapeutic candidates like metformin or pravastatin. However, addressing critical knowledge gaps requires robust longitudinal cohorts and rigorous causal inference to validate complex mechanisms. Furthermore, implementing standardized biomarker protocols remains essential for clinical translation. This review provides a comprehensive framework for precision-based strategies to manage GDM-related fetal overgrowth effectively. Search Strategy. A systematic search of PubMed, Web of Science, and Google Scholar was conducted for literature published up to 2025. The search utilized a combination of the following keywords and their variants: “gestational diabetes mellitus,” “fetal overgrowth,” “macrosomia,” “placental transport,” “insulin resistance,” “mTOR,” “extracellular vesicles,” “microbiome,” and “epigenetics.” Boolean operators (AND, OR) were applied. Priority was given to human clinical studies, meta-analyses, and large cohort studies, with animal and *in vitro* experiments included as mechanistic supplements.

## 1 Introduction

Gestational diabetes mellitus (GDM) is currently one of the most common pregnancy-related metabolic disorders, posing significant risks to both maternal and fetal health. Extensive research has shown that GDM significantly increases the incidence of various adverse perinatal outcomes, with fetal overgrowth being the most common and clinically challenging among them (Usta et al., 2017).

Fetal overgrowth is a heterogeneous clinical phenotype typically defined as abnormally accelerated intrauterine growth velocity beyond physiological expectations. It is commonly operationalized using two related but distinct clinical definitions: macrosomia and large for gestational age (LGA). Macrosomia is typically defined as an absolute birth weight ≥4,000 g or ≥4,500 g, irrespective of gestational age, whereas LGA refers to a birth weight above the 90th percentile for gestational age and sex (Catalano and Shankar, 2017). Therefore, in this review, the term fetal overgrowth is used as an umbrella concept, while macrosomia and LGA are applied according to their specific epidemiological definitions.

**FIGURE 1:**
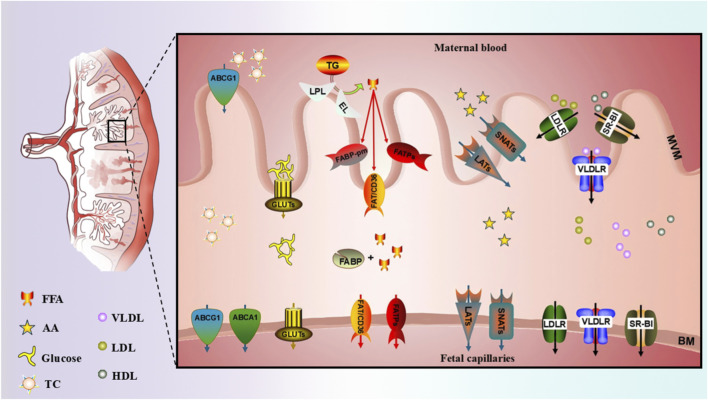
Transport of nutrients closely related to fetal growth in the placenta. The mechanism by which nutrients closely related to fetal growth in the placenta, including fatty acids, amino acids, glucose, and cholesterol, undergo transmembrane transport between maternal blood and fetal capillaries via various transport proteins and receptors on the MVM and BM. Abbreviations: MVM, microvillous plasma membrane; BM, basal plasma membrane; GLUTs, glucose transporters; SNATs, sodium-coupled neutral amino acid transporters; LATs, large neutral amino acid transporters; FATPs, fatty acid transporter proteins; FABP, fatty acid binding protein; TG, triglycerides; LPL, lipoprotein lipase; EL, endothelial lipase; FAT/CD36, fatty acid translocase; FABPpm, plasma membrane fatty acid binding protein; ABCG1, ATP-binding cassette transporter G1; ABCA1, ATP-binding cassette transporter A1; LDLR, low-density lipoprotein receptor; SR-BI, scavenger receptor class B type I; VLDLR, very low-density lipoprotein receptor; FFA, fatty acids; AA, amino acids; TC, total cholesterol; VLDL, very low-density lipoprotein; HDL, high-density lipoprotein; LDL, low-density lipoprotein.

In recent years, understanding of the pathogenesis of GDM-related fetal overgrowth has advanced substantially. Fetal growth depends not only on maternal nutrient availability but also on placental transport efficiency (Figure 1) (Sferruzzi-Perri et al., 2023). The classical Pedersen hypothesis posits that excess maternal glucose in GDM crosses the placenta, induces fetal hyperinsulinemia, and drives overgrowth of insulin-sensitive tissues, resulting in macrosomia (Pedersen, 1954). Building on this model, subsequent studies have highlighted the contribution of placental lipid transport. Szabo et al. proposed that abnormal maternal-fetal fatty acid transfer in GDM promotes fetal adipocyte differentiation, accelerating adipose tissue expansion and increasing birth weight and later obesity risk (Szabo, 2019). The prevailing view suggests that maternal-fetal glucose and lipid metabolic disturbances form the core pathological basis of GDM-related fetal overgrowth. These disturbances promote excessive transplacental transport of nutrients such as glucose and lipids, driving abnormal fetal growth (Chagovets et al., 2025). Importantly, fetal overgrowth reflects not only increased birth weight but also altered body composition, with excessive fat accumulation more strongly associated with adverse perinatal and long-term metabolic outcomes than weight gain alone (Lingwood et al., 2011; Catalano and Shankar, 2017).

Epidemiological data indicate that macrosomia occurs in approximately 12% of normal pregnancies but increases to 15%–45% in those complicated by GDM (Kc et al., 2015). Notably, clinical studies show that fetal overgrowth often persists despite standardized glycemic management and achievement of recommended glucose targets (Mackin et al., 2018; He et al., 2023), suggesting that maternal hyperglycemia, although critical, is not the sole determinant of excessive fetal growth.

Fetal growth in GDM is instead jointly regulated by multiple maternal, placental, and fetal factors, including pre-pregnancy body mass index (BMI), gestational weight gain, fetal sex, parity, and the timing and intensity of glycemic interventions (Luo et al., 2021; Shaat et al., 2024; Li et al., 2025; Maghalian et al., 2025). These factors act through distinct biological pathways but converge on similar growth phenotypes, complicating causal interpretation. In addition, placental vascular function and growth-related signaling pathways contribute to fetal growth regulation (Díaz Del Moral et al., 2021; Lorigo and Cairrao, 2022; Rosario et al., 2023a), while emerging evidence implicates maternal gut microbiota alterations and extracellular vesicle (EVs) -mediated maternal-fetal communication in GDM-related fetal overgrowth (Tkach and Théry, 2016; Miko et al., 2022). Together, these findings extend the traditional glucose-centered framework and help explain why fetal overgrowth may persist despite guideline-compliant glycemic control during pregnancy.

In summary, GDM-related fetal overgrowth is characterized by a high incidence and a multifactorial etiology, making elucidation of its underlying mechanisms and optimization of intervention strategies of considerable clinical and public health importance. However, the deep molecular mechanisms driving this condition have not yet been fully clarified, and current preventive and therapeutic approaches remain to be further refined. In this review, we systematically synthesize the pathological mechanisms and intervention pathways associated with GDM-related fetal overgrowth, integrate recent advances in the field, and highlight key directions for future research.

## 2 Pathogenic mechanisms of GDM-related fetal overgrowth

### 2.1 Abnormal nutrient transport in the placenta

#### 2.1.1 Enhanced placental glucose transport mediated by GLUT1

In GDM, the sustained maternal hyperglycemic environment promotes increased transplacental glucose transport. This excessive glucose is subsequently converted into adipose tissue deposition in the fetus, ultimately leading to the development of macrosomia (Kc et al., 2015). This phenomenon occurs because the fetal pancreas is highly sensitive to fluctuations in circulating glucose concentrations. Exposure to hyperglycemia rapidly induces fetal hyperinsulinemia (Yan Y. S. et al., 2023). Moreover, while the fetus has the intrinsic capacity for gluconeogenesis, this metabolic capability is limited by the delayed expression and activation of phosphoenolpyruvate carboxykinase (PEPCK), an enzyme whose functional activity is typically not observed until the neonatal period (Vargas et al., 2022). Consequently, the conversion of pyruvate to glucose remains functionally limited before birth, rendering the fetus almost entirely dependent on maternally derived glucose via placental transport during this developmental stage (Santos et al., 2023). Studies have demonstrated that when maternal blood glucose levels are ≤120 mg/dL, the incidence of macrosomia is approximately 20%. However, this risk rises significantly to 35% when blood glucose levels reach 160 mg/dL (Kc et al., 2015).

The placental glucose transport process primarily depends on the GLUT gene family, which comprises 12 members. Among these, GLUT1 serves as the key mediator of glucose transport across the placenta and is predominantly expressed in the syncytiotrophoblast (STB). Under normal physiological conditions, GLUT1 expression on the microvillous membrane is markedly higher than on the basal membrane. During weeks 16–22 and 27–30 of gestation, both the expression levels and transport activity of GLUT1 on the basal membrane are significantly increased (Illsley and Baumann, 2020). *Ex vivo* and observational studies of human GDM placentas consistently demonstrate increased basal membrane GLUT1 expression, enhanced glucose uptake, and a positive association with fetal birth weight, influenced by maternal pre-pregnancy BMI (Gaither et al., 1999; Stanirowski et al., 2019), suggesting that upregulated expression of placental GLUT1 may play a critical role in the pathogenesis of GDM-related fetal overgrowth. Studies demonstrate that antenatal exercise interventions induce significant downregulation of placental GLUT1 expression in GDM pregnancies, concurrently attenuating the disorder’s adverse effects on cardiac, hepatic, and renal organogenesis in offspring (Tang et al., 2024). Although this protective effect may not be solely attributed to the downregulation of GLUT1, accumulating evidence indicates that maternal hyperglycemia during pregnancy is strongly associated with fetal overgrowth, adverse perinatal outcomes, and even long-term developmental impairments. Therefore, we propose that epigenetic modifications in placental tissue may represent a key regulatory mechanism, exerting long-term influences on offspring metabolic function development through metabolic programming (Picó et al., 2019). Taken together, the aforementioned human observational studies and *ex vivo* placental experiments consistently indicate a positive association between maternal hyperglycemia, upregulation of placental GLUT1 expression, and fetal birth weight in GDM pregnancies. It is recognized that GLUT1 expression varies with gestational age and maternal pre-pregnancy BMI, both of which may confound the observed associations. Consequently, whether upregulated GLUT1 expression is specifically linked to fetal overgrowth, rather than merely reflecting broader metabolic dysregulation in GDM, remains to be fully elucidated.

#### 2.1.2 Abnormal amino acid (AA) transport in the placenta

Amino acids required for fetal growth and development are primarily utilized in biosynthetic processes such as protein synthesis, carbon storage, and oxidative metabolism, collectively influencing the intrauterine growth rate. The availability of amino acids to the fetus is largely dependent on the placental transport capacity (Vaughan et al., 2017). The human placenta contains more than 15 AA transport systems, among which Systems A and L are the most functionally characterized. Enhanced activity of these AA transport systems is recognized as a key contributor to fetal overgrowth (Shimada et al., 2024).

Placental tissues from women with GDM exhibit significant impairments in amino acid transport function. Increased activity or expression of both System A and System L transporters has been reported in women with GDM and is associated with accelerated fetal growth and fetal overgrowth (Jansson et al., 2006), and studies using primary human trophoblast cells suggest that insulin may enhance AA transport via activation of the Akt and ERK signaling pathways (Castillo-Castrejon et al., 2019). These aberrant placental nutrient transport mechanisms result in sustained elevations of multiple AA levels in umbilical cord blood of fetuses from GDM pregnancies, particularly total branched-chain amino acids (BCAAs), leucine, and valine. These AA not only support placental metabolic activities that promote accelerated fetal growth but are also transported across the placenta into the fetal circulation, leading to disrupted BCAAs metabolism. This metabolic disturbance is not limited to the perinatal period but can persist into childhood (Liu et al., 2023). Abnormalities in BCAAs metabolism are strongly associated with increased cardiac metabolic risk in offspring (Jauhiainen et al., 2021). Macrosomia, a common complication of GDM, is closely linked to an elevated risk of cardiovascular disease (CVD) in adulthood (Contreras-Duarte et al., 2020). These findings suggest that enhanced amino acid transport not only promotes excessive fetal growth but also predisposes macrosomia to long-term CVD risks. Overall, human placental studies indicate that the activity of System A and System L transporters is significantly enhanced in placentas from GDM pregnancies, while *in vitro* experiments further demonstrate that insulin can modulate this transport activity. However, important knowledge gaps remain. How the expression and function of System A and System L transporters dynamically change across gestation, and how they are regulated by maternal metabolic characteristics such as maternal BMI, have not yet been systematically investigated. Consequently, whether enhanced placental AA transport serves as a primary driver of fetal overgrowth or represents a compensatory response secondary to maternal metabolic disturbances, and how it dynamically interacts with maternal metabolic states (e.g., insulin resistance and hyperaminoacidemia), remain to be elucidated through longitudinal studies incorporating gestational age stratification and repeated sampling across pregnancy.

#### 2.1.3 Enhanced placental free fatty acids (FFA) transport and increased fetal fat accumulation

FFA play essential roles in fetal development, including neural differentiation and fat accumulation (Duttaroy and Basak, 2021). In maternal circulation, fatty acids mainly exist as triglycerides (TG), phospholipids, and cholesterol (Du et al., 2024). From approximately the 12th week of gestation, maternal phospholipid, cholesterol, and TG levels progressively increase due to estrogen stimulation and the development of insulin resistance (Ghio et al., 2011). During late pregnancy, enhanced adipose tissue lipolysis accelerates fat mobilization, and the release of endogenous FFA, together with dietary fatty acids and increased hepatic TG synthesis, leads to elevated maternal circulating TG concentrations (Duttaroy and Basak, 2020; Sivan et al., 1999; Xiang et al., 1999).

Although fetal fat has traditionally been considered to derive primarily from glucose conversion, an alternative hypothesis proposes that maternally derived fatty acids transported across the placenta constitute a major source. In GDM, abnormally elevated maternal lipid levels enhance placental fatty acid transport efficiency, increasing both the number and size of fetal adipocytes and promoting fetal overgrowth (Szabo, 2019). TG cannot cross the syncytiotrophoblast (STB) directly; instead, lipoprotein lipase (LPL) and endothelial lipase (EL) localized on placental microvilli hydrolyze maternal TG into FFA (Chassen and Jansson, 2020). LPL further hydrolyzes maternal chylomicron triglycerides (CM-TG) and very low-density lipoprotein triglycerides (VLDL-TG), thereby enhancing placental FFA uptake and fetal adipose accumulation. Consistently, placental lipase activity positively correlates with neonatal fat content and maternal TG levels (Heerwagen et al., 2018). Epidemiological studies show that elevated maternal TG levels in both GDM and non-GDM pregnancies are associated with an increased risk of LGA and macrosomia, with a substantially higher risk observed in women with GDM (Peng et al., 2025). Compared with normal pregnancies, placentas from women with GDM exhibit increased LPL expression, elevated placental TG content, and higher FFA levels in umbilical cord plasma (Balachandiran et al., 2021b), while EL expression is further upregulated in obese women with GDM (Gauster et al., 2011). Enhanced placental LPL and EL activity facilitates the transfer of greater amounts of FFA into the fetal circulation, potentially contributing to GDM-related fetal overgrowth and increasing the risk of macrosomia.

Placental FFA uptake is mediated by specific fatty acid transporters, including fatty acid transport proteins (FATP), fatty acid translocase (FAT/CD36), plasma membrane fatty acid-binding protein (FABPpm), and intracellular fatty acid-binding proteins (FABP). The expression of these transporters is regulated by fatty acid-activated transcription factors such as PPARs, LXR, RXR, and SREBP-1 (Duttaroy and Basak, 2021). Compared with placentas from infants with normal birth weight, placentas associated with macrosomia show significantly higher mRNA and protein expression of PPARα, FABPpm, and FAT/CD36 (Ni et al., 2022). In pregnancies complicated by GDM, dysregulated FABP expression has been linked to excessive fetal lipid accumulation, with placental liver-type fatty acid-binding protein (L-FABP) increased by 64% (Magnusson et al., 2004) and FABP4 and FABP5 significantly upregulated (Scifres et al., 2011). In addition to human placental studies, increased placental expression of FABP2 and FABP3 has been reported in rat models of GDM (Mishra and Kumar, 2023). Elevated glucose levels in placental tissue from GDM pregnancies reduce fatty acid oxidation capacity by approximately 30% while increasing TG accumulation by nearly threefold (Visiedo et al., 2013). Consistently, macrosomic infants born to women with GDM exhibit higher body fat content than those born to mothers with normal glucose tolerance (Martins et al., 2023). However, most available evidence is derived from cross-sectional human placental studies, limiting insights into temporal dynamics and regulatory hierarchies of placental lipid transport. Moreover, pre-pregnancy BMI is associated with altered expression of multiple placental fatty acid transport-related genes (Segura et al., 2017), indicating that maternal obesity and metabolic background factors may interact with these pathways. Overall, current evidence supports a critical regulatory role of placental lipid transport in GDM-related fetal overgrowth, highlighting the need for future studies integrating mechanistic animal models and trophoblast-based systems while controlling for maternal metabolic background.

#### 2.1.4 Enhanced placental cholesterol transport

Cholesterol, as a key structural component of cell membranes and an essential precursor for steroid hormone synthesis, plays an irreplaceable physiological role in fetal growth and development (Chatuphonprasert et al., 2018). Although the fetus possesses endogenous cholesterol synthesis capacity, prior to 19 weeks of gestation, its cholesterol supply primarily depends on maternal provision (Lu et al., 2022). The transport of cholesterol from the maternal to fetal circulation relies on ATP-binding cassette transporter A1 (ABCA1) and G1 (ABCG1) located in STB cells and fetal vascular endothelial cells (Aguilera-Olguín and Leiva, 2022). In addition to direct transport, the placenta also transports cholesterol from the maternal circulation to the fetus by uptake of low-density lipoprotein (LDL), high-density lipoprotein (HDL), and very low-density lipoprotein (VLDL) (Winterhager and Gellhaus, 2017). LDL and HDL in the maternal circulation are taken up by binding to the low-density lipoprotein receptor (LDLR) and scavenger receptor class B type I (SR-BI) on the surface of STB cells, respectively (Aguilera-Olguín and Leiva, 2022). In normal pregnancies, maternal total cholesterol (TC) and TG levels are positively correlated with neonatal birth weight (Kulkarni et al., 2013). A mouse model fed a high-cholesterol diet demonstrated that maternal high-cholesterol intake increases placental cholesterol transport efficiency, leading to lipid deposition in the fetal liver and potentially causing fetal growth and developmental abnormalities (Kuentzel et al., 2022).

Numerous studies have demonstrated that GDM is strongly associated with maternal lipid metabolism disorders. A meta-analysis revealed that, compared to non-GDM pregnancies, GDM patients exhibit significantly elevated serum TG, TC and LDL levels, while HDL levels are decreased (Hu et al., 2021). This lipid dysregulation is clearly linked to adverse perinatal outcomes: elevated TG levels during mid-to-late pregnancy are associated with an increased risk of LGA infants, macrosomia, and neonatal complications, whereas higher HDL levels show a negative correlation with the risk of LGA and adverse neonatal outcomes (Shi et al., 2023). Upregulation of ABCA1 and ABCG1 expression has been observed in human placental endothelial cells isolated from GDM pregnancies (Sun et al., 2018). The protein expression of placental SR-BI is elevated in normal-weight GDM women compared to both healthy controls and overweight/obese GDM subjects. Meanwhile, both mRNA and protein expression levels of LDLR and very low-density lipoprotein receptor (VLDLR) are significantly increased in the placentas of GDM women, regardless of body weight status (Dubé et al., 2013; Fruscalzo et al., 2021). Although the precise mechanisms underlying placental cholesterol and lipoprotein transport in GDM remain incompletely understood, accumulating evidence suggests that disturbances in cholesterol and lipoprotein metabolism may represent a key pathophysiological mechanism contributing to GDM-related fetal overgrowth. It is noteworthy that current research has insufficiently addressed the temporal dynamics of placental cholesterol metabolism, failing to systematically elucidate its regulatory patterns on fetal development across different stages of pregnancy. Additionally, factors such as maternal obesity, dietary structure, and genetic background may influence placental function through pathways like epigenetic modifications, yet existing evidence remains inadequate to unravel the network effects of these complex interactions. Future studies should establish longitudinal research models spanning the entire gestation period, integrating multi-omics technologies and spatial transcriptomics to dynamically dissect the spatiotemporally specific regulatory mechanisms of the placental cholesterol transport system in the pathological progression of GDM.

### 2.2 Fetal growth-related signaling pathways

#### 2.2.1 Regulatory role of the insulin-like growth factor (IGF)

Numerous studies have demonstrated that the IGF system plays a central regulatory role in bodily growth and development, particularly during embryogenesis, the maintenance of metabolic homeostasis, and fundamental biological processes such as cell proliferation and differentiation, where it performs indispensable functions (Díaz Del Moral et al., 2021). The ligands of the IGF system include IGF-I, IGF-II, and insulin, which also contribute significantly to placental development through interactions with their specific receptors (Martín-Estal and Castorena-Torres, 2022).

Compared with those in normal pregnancies, maternal circulating and fetal serum IGF-I concentrations are markedly elevated in pregnancies complicated by GDM. A human prospective observational cohort study showed that maternal plasma IGF-I levels increased by an average of 55.4% between 24–28 and 32–35 weeks of gestation. Moreover, with every 1 standard deviation increase in maternal circulating IGF-I levels, fetal birth weight increased by 75 g, placental weight increased by 20 g, and the risk of macrosomia rose by 1.91-fold (Luo et al., 2012). Notably, lncRNA-SNX17 expression is significantly upregulated in placental tissues from pregnancies affected by GDM-induced macrosomia. Further experiments using human trophoblast cell lines have shown that overexpression of lncRNA-SNX17 suppresses miR-517a expression and elevates IGF-I levels, thereby promoting trophoblast cell proliferation (Guiyu et al., 2022). Furthermore, growth hormone (GH) exerts its effects through the classical GH-insulin-like growth factor-I (GH-IGF-I) axis, influencing pregnancy outcomes in women with GDM. Suppression of the GH-IGF-I axis leads to short stature in offspring, while excessive activation of this axis results in pathological fetal overgrowth (Reynolds et al., 2017; Chesnokova, 2022). Observational human studies have shown that maternal serum GH-V concentrations are significantly higher in GDM pregnancies resulting in LGA infants compared to control pregnancies, and maternal IGF-I levels are also markedly elevated in GDM pregnancies (Liao et al., 2017).

Additionally, elevated IGF-II levels are also detected in the umbilical cord blood of women with GDM (Wang W. J. et al., 2020). Evidence from human observational studies and *in vitro* human trophoblast experiments has indicated that the expression levels of the *IGF-II/H19* gene in the umbilical cord blood of GDM patients are significantly correlated with the development of macrosomia. A potential molecular mechanism may involve the maternal hyperglycemic environment, which can alter the methylation status of the *IGF-II* and *H19* gene promoters in trophoblast cells, thereby increasing the expression of *IGF-II/H19* (Su et al., 2016; Zhang Q. et al., 2022). Furthermore, studies in GDM mouse models have demonstrated that epigenetic changes in *IGF-II/H19* can be inherited across generations (Ding et al., 2012). Dysregulation of this epigenetic control mechanism may contribute to fetal overgrowth, with potential transgenerational effects.

IGF activity is regulated not only by the availability of IGF receptors but also by the insulin-like growth factor-binding protein family (IGFBPs 1–6), which can bind to both IGF-I and IGF-II. In most cases, IGFBPs inhibit the biological actions of IGF through competitive binding mechanisms (Bach, 2018). IGFBP-7, also known as insulin-like growth factor-binding protein-related protein 1 (IGFBP-rP1), exhibits a higher affinity for insulin than for IGF and has been shown to interact with IGF-1R. IGFBP-rP1 functions as an IGF antagonist by directly binding to the receptor and inhibiting IGF-1R activation (Artico et al., 2021). Studies have reported that the levels of IGFBP-1, IGFBP-2, IGFBP-3, and IGFBP-rP1 are significantly reduced in the umbilical cord blood from pregnancies complicated by GDM. Moreover, IGFBP-1 and IGFBP-2 show a negative correlation with fetal birth weight (Lappas, 2015). These findings suggest that fetal overgrowth may result from the downregulation of IGFBP and IGFBP-rP1 expression, which increases IGF bioavailability and consequently accelerates fetal growth and development abnormally. Current evidence linking the IGF system to GDM-related fetal overgrowth is derived primarily from observational human studies demonstrating correlations between circulating or umbilical cord IGF levels and fetal growth outcomes. Although trophoblast cell models indicate that IGF signaling regulates placental gene expression and cellular proliferation, direct mechanistic evidence from animal intervention studies or integrated placental functional models is still lacking to confirm whether IGF actively drives placental nutrient transport and fetal metabolic programming *in vivo*. Therefore, future research should integrate spatially and temporally resolved placental tissue analyses, conditional genetic animal models, and longitudinal clinical cohort data to clarify the specific causal role of IGF signaling in GDM-related fetal overgrowth and its potential translational relevance.

#### 2.2.2 mTOR signaling pathway as a central regulator of placental nutrient transport

The placental mTOR signaling pathway plays a central role in regulating fetal growth (Rosario et al., 2023a). The mTORC1 signaling pathway primarily promotes protein synthesis through two distinct mechanisms: first, it regulates the function of the eukaryotic initiation factor 4E (eIF4E) by phosphorylating the 4E-binding protein (4E-BP); second, it phosphorylates ribosomal protein S6 kinase 1 (S6K1), which in turn modulates the activity of eIF4B and enhances ribosomal biosynthesis capacity (Panwar et al., 2023). Placental tissues from macrosomic offspring of GDM mothers exhibited significantly enhanced mTORC1 signaling pathway activity (Sati et al., 2016). Furthermore, study based on human-derived placental tissues has further demonstrated that phosphorylation levels of the two aforementioned mTORC1 downstream targets (S6K1 and 4EBP-1) exhibit positive correlations with neonatal birth weight (Jansson et al., 2013). Although no direct evidence currently confirms that the mTORC1 signaling pathway causes macrosomia by promoting protein synthesis, its excessive activation may represent a potential contributing factor to fetal overgrowth.

As an E3 ubiquitin ligase, neuronal precursor cell expressed developmentally downregulated 4–2 (Nedd4-2) catalyzes protein ubiquitination leading to proteasomal degradation. Using primary human trophoblast models, Rosario F.J et al. first demonstrated a novel regulatory relationship between the mTORC1 signaling pathway and ubiquitination-a common post-translational modification. Their findings revealed that mTORC1 activation suppresses Nedd4-2 expression, thereby reducing ubiquitination of placental AA transporters. This subsequently enhances the transport capacity of SNAT2 and LAT1, ultimately promoting amino acid uptake in trophoblast cells. These discoveries may provide new perspectives for exploring mechanisms underlying fetal overgrowth (Rosario et al., 2016).

**FIGURE 2:**
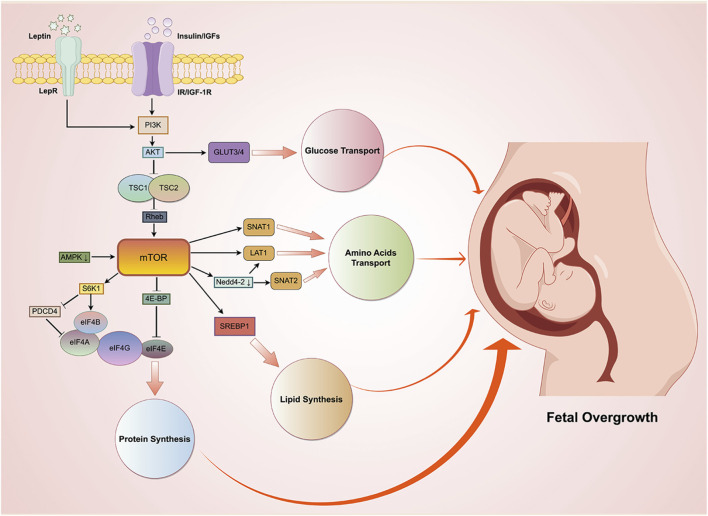
Schematic diagram of mTOR signaling pathway regulation in fetal overgrowth. In patients with GDM, elevated insulin, IGF-I, and leptin promote protein and lipid synthesis by activating the mTOR pathway and enhance the placenta’s transport function for amino acids and glucose, thereby collectively leading to fetal overgrowth. Abbreviations: TSC1, tuberous sclerosis complex 1; TSC2, tuberous sclerosis complex 2; Rheb, ras homolog enriched in brain; AMPK, AMP-activated protein kinase; PDCD4, programmed cell death protein 4; eIF4, eukaryotic translation initiation factor 4; SREBP1, sterol regulatory element-binding protein 1.

A human prospective cohort study has demonstrated that reduced AMPK phosphorylation in placental tissue is associated with activation of the mTORC1 signaling pathway, a phenomenon that is significantly linked to fetal overgrowth (Keleher et al., 2020). Among 50 GDM patients with blood glucose levels maintained within the normal range, 23 delivered macrosomia and 27 delivered infants with normal birth weight. Analyses of placentas from these pregnancies revealed a significant positive correlation between placental IGF-I activity and mTOR signaling pathway activity, as well as a negative correlation between AMPKα phosphorylation levels and fetal birth weight (Shang and Wen, 2018). Compared with placentas from normal pregnancies, those from GDM pregnancies complicated by LGA infants exhibited lower AMPKα phosphorylation levels and higher mTOR phosphorylation levels (Hung et al., 2021). In placentas from small-for-gestational-age (SGA) fetuses, LAT1 expression was decreased (Chen et al., 2015), whereas LAT1 expression was upregulated in LGA placentas. LAT1 is encoded by the *SLC7A5* gene, and studies have shown that overexpression of *SLC7A5* in primary human trophoblast cells not only significantly enhances systemic L-amino acid transport function but also increases systemic A-amino acid transport efficiency by 38%. Furthermore, LAT1 upregulation promotes activation of the mTOR signaling pathway by suppressing AMPK signaling activity (Rosario et al., 2023b). These findings collectively demonstrate intricate interconnections between the mTOR signaling pathway and fetal growth-regulatory networks, revealing its central regulatory role in governing fetal development (Figure 2). Importantly, dysregulated activation of mTOR-related pathways may constitute the pivotal mechanism underlying GDM-induced fetal overgrowth. However, current evidence on the role of the mTOR signaling pathway in placental function regulation in GDM primarily originates from observational studies or *ex vivo* placental models, which limits interpretability to some extent. It is important to note that placental mTOR pathway activity is highly dependent on dynamic regulation by multiple factors, including maternal nutrient supply, hormonal levels, and oxidative stress. Existing experimental systems struggle to accurately replicate this complex and time-varying physiological environment. Additionally, mTOR signaling is highly sensitive to cellular context, and significant differences may exist across different trophoblast subtypes, developmental stages, and fetal sex. However, these critical variables are often inadequately accounted for in current research. Future studies urgently need to integrate cell-specific genetic models, dynamic functional analyses, and longitudinal study designs spanning different gestational stages to more precisely dissect the specific roles of mTOR signaling in placental adaptive regulation and fetal growth control.

### 2.3 Placental cellular and vascular dysfunction

#### 2.3.1 Imbalance in trophoblast proliferation, autophagy, and apoptosis

Apoptosis is a naturally occurring process in placental cells and plays a crucial role in placental growth, development, and aging. Maintaining a balance between trophoblast cell apoptosis and proliferation is essential for normal placental development (Xie et al., 2024). In patients with GDM, an increased number of trophoblast cells and enhanced proliferation activity have been observed in placental tissues, accompanied by a reduction in apoptosis (Duan et al., 2023), which may contribute to increased placental weight. Elevated placental weight has been significantly associated with the development of LGA infants (Hung et al., 2020). The epidermal growth factor receptor (EGFR) serves as a key regulator of cell proliferation, human growth and development, and embryogenesis. It is highly expressed in placental tissues and plays a pivotal role in regulating placental growth and development through its involvement in critical physiological processes such as trophoblast cell fusion (Clemente and Bird, 2023). In mouse experiments, it has been confirmed that after excluding the interference of maternal blood sugar and blood lipids on nutrient supply, EGFR can still significantly promote the proliferation of placental sponge trophoblast cells and lead to an increase in placental volume (Dackor et al., 2009). Recent studies have identified a positive feedback regulatory loop involving glycoprotein hormone alpha subunit (CGA), EGFR, and transcription factor GATA binding protein 2 (GATA2), which contributes to the pathological proliferation of placentas in GDM-induced macrosomia (Xu et al., 2024). Moreover, increased expression of proliferating cell nuclear antigen (PCNA) has been observed in placentas associated with GDM-induced macrosomia. *In vitro* studies have demonstrated that a high-glucose environment significantly stimulates trophoblast cell proliferation and activates the phosphorylation of the ERK1/2 signaling pathway. Notably, specific inhibitors of ERK1/2 can effectively suppress this hyperproliferative response induced by high glucose levels (Zheng et al., 2022). Additionally, elevated expression of FABP4 has been observed in placentas of macrosomic infants born to mothers with GDM. Importantly, FABP4 has been demonstrated to enhance the proliferative and migratory capacities of human trophoblast cells (Yan et al., 2016; Yang et al., 2020).

In addition to pathological proliferation, emerging evidence suggests that the increased placental weight and fetal overgrowth observed in patients with GDM may be attributed to dysregulation of gene expression and protein synthesis related to autophagy and apoptosis in placental tissues. These molecular alterations can impair autophagy and reduce apoptosis in trophoblast cells, ultimately contributing to placental tissue remodeling. A decrease in the levels of autophagy- and apoptosis-related proteins has been observed in placentas from GDM pregnancies complicated by LGA fetuses. *In vitro* cell culture studies have further confirmed that a high-glucose environment can impair autophagy and suppress apoptosis in trophoblast cells (Hung et al., 2020). MicroRNAs also play a regulatory role in this process. Research using plasma-derived exosomes from GDM patients and trophoblast cell models has revealed that the miR-99 family inhibits autophagy in GDM placental trophoblast cells by upregulating myotubularin-related protein 3 (MTMR3) expression (Liu and Lv, 2025). Another study revealed that elevated expression of matrix metalloproteinases (MMPs) in the placentas of GDM patients enhances the expression of CSF3R splice variants and activates the PI3K/Akt signaling pathway, thereby reducing trophoblast cell apoptosis (Zhang Y. et al., 2024). The dual pathological alterations characterized by aberrant proliferation of placental trophoblast cells accompanied by suppressed autophagy and diminished apoptosis may constitute a critical pathogenic mechanism underlying the development of GDM-induced macrosomia. It should be particularly noted that current research on the imbalance of trophoblast proliferation/apoptosis and the development of macrosomia in GDM primarily relies on *in vitro* cell models treated with high glucose or postpartum placental samples. These models struggle to replicate the dynamic microenvironmental changes at the maternal-fetal interface during pregnancy. There is a notable lack of longitudinal studies on the temporal evolution of trophoblast behavior across different stages of pregnancy, making it difficult to determine whether the observed cellular phenotypes represent a persistent pathological state or a stage-specific adaptive response. Future studies should establish three-dimensional placental organoid models capable of simulating the dynamic fluctuations in blood glucose levels during pregnancy, combined with live-cell imaging techniques, to analyze the causal relationship between abnormal trophoblast behavior and fetal growth trajectories in both spatial and temporal dimensions.

#### 2.3.2 Abnormal placental angiogenesis and compromised barrier function

Placental angiogenesis is a tightly regulated dual-phase process governed by the balance between pro-angiogenic and anti-angiogenic factors. Altered placental vascular function can impair fetal development and increase the long-term risk of CVD in offspring (Lorigo and Cairrao, 2022). This process is dynamically regulated by vascular active factors, particularly vascular endothelial growth factor (VEGF), which plays a central role in placental vascular development, especially during early pregnancy (Huang et al., 2021). Structural analyses have demonstrated that placentas from women with GDM exhibit enhanced vascular perfusion and maternal-fetal exchange capacity, characterized by increased villous surface area, higher numbers of small villi and villous vessels, increased capillary density, branching, and surface area (Nanobashvili et al., 2018; Yao et al., 2023; Moreli et al., 2024). These structural adaptations may represent a key mechanism contributing to fetal overgrowth in a hyperglycemic intrauterine environment.

The VEGF family includes VEGF-A, -B, -C, -D, -E, -F, and placental growth factor (PIGF), which exert their effects through VEGF receptor-1 (VEGFR-1/Flt-1), VEGFR-2 (KDR/Flk-1), and VEGFR-3 (Flt-4) (Melincovici et al., 2018). VEGFR-2, predominantly expressed in endothelial cells and their precursors, mediates the major pro-angiogenic effects of VEGF due to its strong tyrosine kinase activity, promoting endothelial proliferation, migration, and vessel formation (Wang X. et al., 2020). In contrast, VEGFR-1 exhibits high ligand affinity but weak kinase activity, functioning primarily as a negative regulator by competitively binding VEGF-A and limiting VEGFR-2 activation during early vascular development (Huang et al., 2021). In placentas from women with GDM, Flt-1 mRNA and protein levels are significantly reduced, whereas KDR protein expression is abnormally elevated (Troncoso et al., 2017). Immunolocalization studies further show that in mild hyperglycemia, VEGF and VEGFR-2 are strongly expressed in endothelial and trophoblast cells, while VEGFR-1 expression remains weak. In contrast, in GDM placentas, VEGFR-1 is strongly expressed in both vascular and trophoblastic cells, whereas VEGF and VEGFR-2 expression is markedly reduced in placental capillary endothelial cells (Pietro et al., 2010). These findings suggest that intrauterine hyperglycemia initially induces mild hypoxia and compensatory angiogenesis, whereas severe metabolic dysregulation leads to pronounced placental hypoxia and impaired functional capillary formation, although the underlying mechanisms remain incompletely understood (Pietro et al., 2010).

In addition to angiogenic signaling, placental barrier integrity is disrupted in GDM patients. Key adhesion and junctional proteins, including vascular endothelial cadherin, β-catenin, occludin, and zonula occludens-1, are significantly downregulated in GDM placentas (Babawale et al., 2000; Villota et al., 2021), potentially impairing maternal-fetal substance exchange. Connexin 43 (Cx43), a regulator of monocyte-endothelial adhesion, is markedly upregulated in umbilical vein endothelial cells from GDM pregnancies, activating the PI3K/AKT/NF-κB pathway and increasing ICAM-1 and VCAM-1 expression (Zhang et al., 2021). This promotes excessive monocyte-endothelial adhesion, amplifies inflammatory signaling, and further aggravates vascular dysfunction (Khambule and George, 2019), thereby compromising placental barrier integrity and facilitating excessive nutrient transfer to the fetus.

Insulin also plays a crucial regulatory role in placental vascular biology. It exerts its physiological effects through activation of insulin receptors, which include two subtypes-IR-A and IR-B-both of which are expressed in fetal placental endothelial cells, such as human umbilical vein endothelial cells (HUVECs) and human placental microvascular endothelial cells (hPMECs) (Westermeier et al., 2016). Activation of the insulin signaling pathway via the PI3K/Akt cascade enhances the expression and activity of endothelial nitric oxide synthase (eNOS), leading to increased production of nitric oxide (NO) (Subiabre et al., 2020). Current evidence suggests that in HUVECs derived from GDM, both IR-A and IR-B expression levels are significantly elevated, accompanied by enhanced Akt signaling activity, as well as increased eNOS activity and NO synthesis (Westermeier et al., 2015; Vedika et al., 2023). eNOS plays a pivotal role in promoting fetal growth and development and enhancing placental perfusion (Krause, 2021).

**FIGURE 3:**
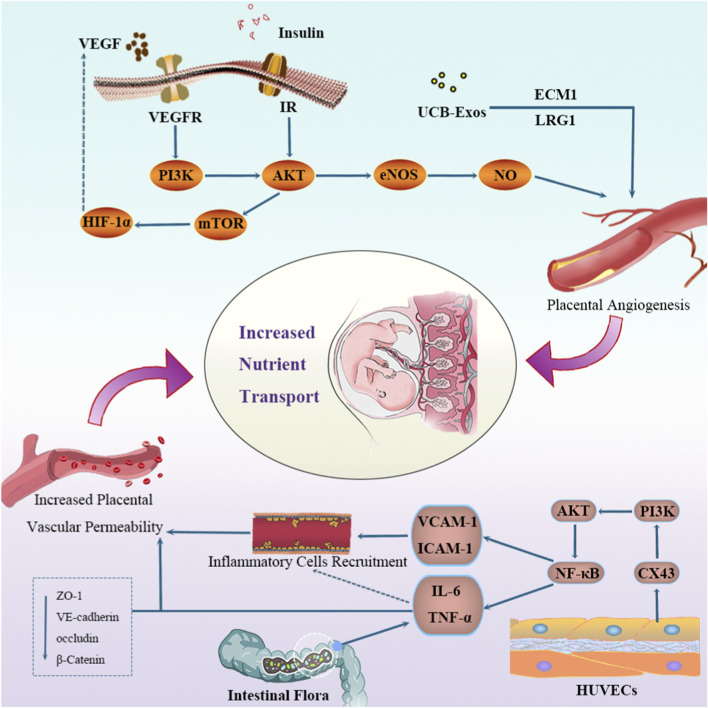
Mechanisms underlying the aberrant increase in nutrient transport across the placenta in GDM. In patients with GDM, elevated VEGF and insulin synergize with umbilical cord blood-derived exosomal LRG1 and ECM1 to collectively promote excessive placental vascular proliferation. On the other hand, highly expressed Cx43 in umbilical vein endothelial cells upregulates ICAM-1 and VCAM-1 expression, enhancing abnormal monocyte-endothelial cell adhesion and stimulating the secretion of inflammatory factors IL-6 and TNF-α. Simultaneously, the expression levels of adhesion proteins VE-cadherin, β-catenin, occludin, and ZO-1 in the placenta are significantly reduced. These changes collectively lead to abnormally increased placental vascular permeability. Consequently, the excessive transport of nutrients from the GDM placenta to the fetus accelerates fetal growth. Abbreviations: PI3K, phosphoinositide 3-Kinase; Akt, protein kinase B; HUVECs, human umbilical vein endothelial cells; eNOS, endothelial nitric oxide synthase; HIF-1α, hypoxia-inducible factor 1-Alpha; NF-κB, nuclear factor Kappa-light-chain-enhancer of activated B cells; mTOR, mammalian target of rapamycin; ECM1, extracellular matrix protein 1; LRG1, leucine-rich Alpha-2-Glycoprotein 1; ZO-1, zonula occludens-1; VE-cadherin, vascular endothelial cadherin; VEGF, vascular endothelial growth factor; VCAM-1, vascular cell adhesion molecule-1; ICAM-1, intercellular adhesion molecule-1.

Cellular responses to hypoxia are mediated largely by hypoxia-inducible factor-1 (HIF-1), composed of HIF-1α and HIF-1β, which regulates angiogenesis, glycolysis, and cell proliferation (Yang et al., 2021). In GDM pregnancies, fetal hyperinsulinemia and insulin-induced eNOS activation increase metabolic demand and oxygen consumption, predisposing the fetus to hypoxia (Desoye and van Poppel, 2015). Both eNOS activation and hypoxic stress contribute to the stabilization and transcriptional activation of HIF-1, which subsequently upregulates VEGF expression and promotes angiogenesis (Krause, 2021). In women with GDM, the combined actions of VEGF, insulin signaling, and HIF-1 coordinately regulate placental angiogenesis and barrier function, thereby enhancing nutrient transport to the fetus and influencing both placental and fetal growth and development (Figure 3). However, it remains important to recognize that many of the observed structural and molecular changes-such as enhanced vascular density, upregulated VEGF signaling, and altered junctional protein expression-may represent compensatory adaptations aimed at maintaining adequate nutrient and oxygen delivery under metabolic stress, rather than purely pathological processes. Moreover, most existing studies rely on expression-level analyses of angiogenic factors, adhesion molecules, or signaling components, while direct functional assessments of placental permeability, transendothelial transport capacity, or barrier integrity remain limited. As a result, it is still unclear to what extent these molecular alterations translate into quantitative changes in nutrient flux across the maternal-fetal interface. The frequent coexistence of enhanced angiogenesis with impaired barrier organization further suggests a complex balance between adaptive vascular expansion and compromised structural integrity. Future studies should therefore integrate functional permeability assays, *in vivo* or *ex vivo* placental perfusion models, and quantitative measurements of nutrient transfer to distinguish adaptive angiogenic remodeling from maladaptive barrier disruption. With the continuous advancement of research, progress continues to be made in exploring the molecular mechanisms of GDM-related fetal overgrowth. Table 1 summarizes the relevant research results in recent years.

**TABLE 1:** Research of molecular mechanisms associated with GDM-related fetal overgrowth (last 5 years).

Date	Model	Molecular	Signaling pathway	Functional effects/Findings	References
2024	GDM women with macrosomia placentas	​	CGA-EGFR-GATA2	Promoted STBs proliferation	Xu et al. (2024)
2022	GDM women with macrosomia placentas/BeWo cells	PCNA↑	ERK1/2	Promoted trophoblast cell proliferation	Zheng et al. (2022)
2021	maternal blood/umbilical cord blood/placentas from GDM women	Adiponectin↓, IGF-I↑	Insulin/IGF-I	Increased GLUT-1 expression	Balachandiran et al. (2021a)
2020	GDM women with LGA placenta/CTBs	Beclin-1↓, DRAM↓, M30↓, Cleaved PARP↓, Ki-67↑, Bcl-xL↑, Bak↓, Bad↓	​	Decreased autophagic activity and apoptosis in trophoblast cells	Hung et al. (2020)
2021	GDM women with LGA placenta/CTBs	p-Akt↑, p-AMPKα↓, p-mTOR↑	mTOR	Promoted protein synthesis, accelerated trophoblast cell proliferation, inhibited apoptosis and autophagy	Hung et al. (2021)
2022	GDM women with macrosomia placentas/HTR-8/SVneo cells	lncRNA-SNX17↑	MiR-517a/IGF-I	Promoted trophoblast cell proliferation	Guiyu et al. (2022)
2025	maternal blood/placentas from GDM women/HFD mouse model	Gal-9↑	​	Increased proliferation, decreased apoptosis and impaired autophagy in trophoblast cells	Albuayjan et al. (2025)
2023	placentas from GDM women	LRG1↑, ECM1↑	​	Promoted placental pathological angiogenesis	Yao et al. (2023)
2025	maternal blood/placentas from GDM women	MiR-99 family↓, MTMR3↑	​	Inhibited trophoblast autophagy and exacerbated insulin resistance	Liu and Lv (2025)
2024	placentas from GDM women	MMPs↑, CSF3R↑	PI3K/Akt	Increased proliferation and decreased apoptosis of trophoblast cells	Zhang et al. (2024c)
2023	GDM rat model	FABP2↑, FABP3↑, Slc27a3↑	​	Facilitated transfer of LCPUFA to the fetus	Mishra and Kumar (2023)
2023	placentas from GDM women/HTR-8/SVneo cells	​	TXNIP-NF-κB-STAT3	Accelerated migration and invasion of trophoblast cells	Sa et al. (2023)
2022	placentas from GDM women/GDM mouse model/HUVECs and MUVECs	Chemerin↑, VEGF↑	​	Promoted proliferation, migration and tube formation of umbilical vein endothelial cells	Zhang et al. (2022a)
2021	maternal blood/placentas from GDM women/FpECs	HMOs↑	​	Promoted placental pathological angiogenesis	Hoch et al. (2021)
2021	GDM-hAMSCs	TGFBR1↑, VEGFA↑, FGFR2↑, SERPINE1↑	​	Enhanced angiogenic capacity of hAMSC	Klid et al. (2021)
2021	placentas from GDM women/FpECAds and HUVECs	VEGF↑	Succinate-SUCNR1 axis	Promoted placental pathological angiogenesis	Atallah et al. (2021)
2021	Maternal blood/umbilical cord blood/HTR-8/SVneo cells/GDM mouse model	TMAO↑	​	Inhibited the formation of NETs and enhanced trophoblast cell viability, migration, invasion and angiogenesis	Lin et al. (2021)
2021	placentas from GDM women/HTR-8/SVneo cells/STBs	MiR-9↓, MiR-22↓	​	Increased expression of GLUT1 and HK2	Song et al. (2021)
2021	placentas from GDM women	IMPA1↓, SLC5A11↓, SLC2A13↓	​	Decreased inositol	Pillai et al. (2021)
2020	placentas from GDM women/umbilical cord blood	AQP3↓, APN↓	​	Disordered lipid homeostasis in placenta	Zhang et al. (2020)
2024	placentas from GDM women/HTR-8/SVneo cells	Vitronectin↑	JNK	Insulin resistance increased	Ju et al. (2024)
2024	maternal blood/GDM women with macrosomia umbilical cord blood/JEG-3 and HTR8/SVneo cells	Hsa_circ_0024838↑	MiR-543/HIF1A axis	Promoted the vitality of trophoblast cells	Liu et al. (2024)

## 3 Gut microbiota and extracellular vesicles: emerging regulators of the maternal-fetal environment and communication

### 3.1 Regulatory role of gut microbiota

Recent studies have confirmed that maternal gut microbiota and its metabolites can be transferred to the fetus via the placental route during pregnancy, thereby exerting regulatory effects on fetal growth and development (Miko et al., 2022). Dysbiosis of the gut microbiota can alter microbial composition, compromise intestinal barrier function, and promote the release of pro-inflammatory cytokines (Edwards et al., 2017). Importantly, such microbial alterations do not occur in isolation but are closely associated with maternal metabolic abnormalities, including adiposity, hyperglycemia, and insulin resistance (Liu et al., 2019). This suggests that gut microbiota changes typically arise within the context of systemic metabolic dysregulation rather than as independent events.

Clinical trials have demonstrated that women with GDM exhibit a reduction in beneficial gut bacterial genera alongside relative enrichment of potentially pathogenic bacteria, accompanied by elevated levels of inflammatory factors such as TNF-α, IL-17, and IL-6 (Ding et al., 2021). Similar patterns of microbial imbalance have also been observed in neonates born to mothers with GDM. Moreover, transplantation of fecal microbiota from neonates of GDM pregnancies into mice has been shown to induce systemic inflammation and disrupt intestinal barrier integrity in the recipient mice (Hu et al., 2025), providing mechanistic support for a link between gut microbiota and inflammatory activation. In addition, evidence from GDM mouse models and *in vitro* cell-based experiments indicates that GDM-associated gut microbiota dysbiosis promotes polarization of adipose tissue macrophages toward a pro-inflammatory M1 phenotype, thereby exacerbating insulin resistance (Li et al., 2024). Collectively, these findings suggest that gut microbiota alterations may indirectly amplify maternal insulin resistance through sustained activation of inflammatory signaling. As insulin resistance worsens, maternal glucose and lipid metabolic homeostasis becomes further disrupted, creating a persistently elevated nutritional exposure for the developing fetus.

A clinical study has further shown that at 38 weeks of gestation, the abundance of Bilophila is positively associated with LGA. However, this association is markedly attenuated after adjustment for maternal pre-pregnancy BMI and gestational weight gain, indicating that such microbial features may primarily reflect maternal metabolic burden rather than acting as independent drivers of fetal overgrowth. In contrast, genera such as *Acinetobacter* and *Aeromonas* remain stably and negatively associated with LGA after multivariable adjustment (Pašić et al., 2025), suggesting that they may participate in modulating the fetal growth environment under specific metabolic conditions.

Interventional studies provide additional supportive evidence. Animal experiments have demonstrated that probiotic supplementation can improve maternal insulin resistance (Salles et al., 2020). Consistently, clinical studies indicate that probiotic interventions partially ameliorate glucose and lipid metabolic disturbances in women with GDM, leading to reductions in fasting plasma glucose, fasting serum insulin, TC, and hemoglobin A1c (HbA1c) (Mu et al., 2023). Notably, a randomized controlled trial reported that probiotic supplementation in GDM pregnancies was associated with reduced neonatal birth weight and a lower incidence of macrosomia (Sahhaf Ebrahimi et al., 2019), suggesting that modulation of gut microbiota may indirectly improve fetal growth outcomes.

Overall, current evidence supports a role for gut microbiota in the development of GDM-related fetal overgrowth; however, its effects appear to be highly dependent on maternal inflammatory status, the degree of insulin resistance, and the broader metabolic context. Gut microbiota alterations are therefore more likely to function as amplifiers or modulators of maternal metabolic dysfunction rather than as a primary, independent pathogenic trigger. Future studies integrating longitudinal designs, functional experiments, and stratified analyses will be essential to more clearly delineate the indicative versus causal roles of gut microbiota in GDM-related fetal overgrowth and to assess their potential as therapeutic targets.

### 3.2 Extracellular vesicle-mediated maternal-fetal communication

EVs are nanoscale membrane-bound particles released by multiple cell types. They contain a variety of bioactive molecules, including proteins, mRNA, and microRNA, and play critical roles in intercellular communication, participating in a wide range of physiological and pathological processes (Tkach and Théry, 2016). Among them, exosomes are typically defined as small extracellular vesicles (sEVs) with a diameter of less than 150 nm. The terms “exosomes” and “sEVs” are often used interchangeably in the literature.

During pregnancy, EVs are actively released from diverse maternal and placental tissues, such as adipose tissue, liver, pancreas, skeletal muscle, and the placenta itself. Their concentration, composition, and functional characteristics undergo dynamic shifts in response to gestational age and maternal metabolic health. These regulated changes position EVs as promising candidate biomarkers for a range of pregnancy-related complications (Jayabalan et al., 2017; Hessvik and Llorente, 2018; Théry et al., 2018). In maternal-fetal communication, the placenta plays a pivotal “bidirectional regulatory” role: it not only actively releases EVs into both maternal and fetal circulations but also selectively internalizes maternally derived EVs, thereby mediating the transplacental transfer of maternal or exogenous vesicles into the fetal compartment (Chiarello et al., 2018; Buca et al., 2020; Paul et al., 2023). This unique property provides a structural basis for EV-mediated transmission of maternal metabolic signals to the fetus.

Previous studies have demonstrated that EVs themselves possess the capacity to regulate metabolic homeostasis. For example, EVs derived from human adipose tissue can induce insulin resistance in hepatocytes and skeletal muscle cells through modulation of the Akt signaling pathway (Kranendonk et al., 2014), suggesting that EVs may act as carriers of metabolic signals involved in systemic insulin sensitivity regulation. In the context of GDM, both the quantity and functional characteristics of EVs are markedly altered. Clinical studies have shown that during early pregnancy (11–14 weeks of gestation), plasma exosome concentrations in women who subsequently develop GDM are significantly elevated, reaching approximately twofold higher levels observed in normal pregnancies. Further *in vitro* experiments demonstrated that exosomes isolated from the peripheral blood of women with GDM exhibit clear biological activity, capable of inducing the release of pro-inflammatory cytokines from endothelial cells (Salomon et al., 2015). These findings suggest that, under GDM conditions, EVs may contribute to the amplification of inflammatory signaling, indirectly aggravate maternal insulin resistance, and thereby increase fetal exposure to a hyperglycemic environment.

In addition, a mechanistic study isolated sEVs from the plasma of healthy pregnant women and women with GDM at 24–28 weeks of gestation and intravenously injected them into late-gestation mice for four consecutive days. The results showed that sEVs derived from GDM pregnancies induced impaired glucose tolerance in pregnant mice, whereas sEVs from healthy pregnancies significantly enhanced glucose-stimulated insulin secretion in pancreatic cells, leading to elevated fasting insulin levels. Notably, this compensatory effect was absent in mice treated with GDM-derived sEVs (James-Allan et al., 2022). These findings indicate that sEVs may play a specific role in regulating maternal glucose metabolism during pregnancy, and that functional alterations in GDM-derived sEVs may contribute to the development of GDM and indirectly influence fetal growth through changes in the maternal metabolic environment.

Functionally, both animal and *in vitro* studies further support a potential role for EVs in regulating placental and fetal development. Compared with normal pregnancy, exosomes derived from the umbilical cord blood of GDM pregnancies induced abnormally enhanced placental vascularization in mouse models. This effect was likely associated with enrichment of specific exosomal proteins, particularly leucine-rich α-2 glycoprotein 1 and extracellular matrix protein 1, both of which are closely involved in angiogenic processes (Yao et al., 2023). These findings suggest that EVs may indirectly alter the fetal nutrient supply environment by modulating placental vascular remodeling.

Evidence linking EVs to fetal growth outcomes is also accumulating. Studies have shown that among EVs derived from adipose tissue in normal glucose tolerance and GDM groups, 54 miRNAs exhibit significant differential expression. EVs derived from the GDM group markedly promote glucose uptake in trophoblast cells, and further functional analyses revealed that miR-515-5p not only enhances glucose uptake but also suppresses abnormal increases in pro-inflammatory cytokine production in trophoblasts. Mechanistically, *in vivo* administration of EVs derived from normal glucose tolerance adipose tissue and loaded with miR-515-5p into pregnant mice was associated with fetal body weight and blood glucose levels (Jayabalan et al., 2025), suggesting that specific miRNAs may regulate fetal metabolism and growth through EV-mediated maternal-fetal communication.

Consistent with these experimental findings, human clinical studies have demonstrated that exosomes isolated from the umbilical cord blood of LGA born to mothers with GDM exhibit significant transcriptomic alterations. Differentially expressed genes were predominantly enriched in biological pathways closely related to growth and metabolism, including the PI3K/Akt signaling pathway, insulin resistance, glycerol metabolism, fatty acid degradation, and the mTOR signaling pathway. Further analysis of peripheral blood exosomes identified growth differentiation factor 3 and lncRNA AC006064.4 as potential predictive biomarkers. When assessed at 24–28 weeks of gestation and combined with maternal age, fasting plasma glucose, and 2-h postprandial glucose levels, these biomarkers demonstrated good predictive performance for the occurrence of LGA (Yuan et al., 2022). Collectively, these findings suggest that the molecular cargo carried by EVs not only reflects maternal metabolic status but may also integrate maternal metabolic signals with placental responses, dynamically contributing to regulatory networks underlying GDM-related fetal overgrowth.

Taken together, current evidence indicates that EVs involved in GDM-related fetal overgrowth are primarily derived from maternal peripheral blood and umbilical cord blood, with functional cargo dominated by non-coding RNAs and metabolism-related proteins. Functionally, these EVs may indirectly influence fetal growth trajectories by modulating maternal insulin sensitivity, placental vascular remodeling, and key metabolic signaling pathways. However, it should be noted that most studies on EVs remain exploratory, and direct causal evidence linking EVs to GDM-related fetal overgrowth is still limited. Moreover, confounding factors such as maternal BMI, inflammatory status, and gestational age, as well as methodological heterogeneity in EVs isolation and characterization, further constrain their clinical reproducibility as diagnostic or predictive tools. Therefore, although EVs represent a promising avenue for elucidating the mechanisms of GDM-related fetal overgrowth and developing potential biomarkers, their clinical application will require further standardization and rigorous functional validation.

## 4 Epigenetic modifications and long-term health risks in GDM-related fetal overgrowth

The Developmental Origins of Health and Disease (DOHaD) theory posits that exposure to environmental factors during early life, particularly within the intrauterine environment, can shape developmental trajectories and exert long-lasting effects on health outcomes and disease susceptibility later in life (Saffery and Novakovic, 2014). Early embryonic development is accompanied by extensive and dynamic epigenetic reprogramming (Cantone and Fisher, 2013); consequently, abnormal metabolic, inflammatory, and hormonal conditions during pregnancy may influence offspring physiological and metabolic phenotypes through intrauterine programming mechanisms (Barker, 1998; Fernandez-Twinn et al., 2019). Within this framework, the long-term impact of GDM on fetal development and offspring health has emerged as a major research focus, with accumulating evidence suggesting that its underlying mechanisms are closely linked to metabolic dysregulation and epigenetic alterations (Symonds et al., 2009).

Current research has gradually revealed that epigenetic mechanisms play a central role in the regulation of GDM-related fetal growth. Clinical studies have demonstrated that maternal hyperglycemic exposure can induce alterations in placental DNA methylation patterns (Cardenas et al., 2018), and that these changes are significantly associated with fetal birth weight (Küpers et al., 2019). Multiple investigations have further identified GDM-specific DNA methylation signatures and proposed their potential utility as molecular biomarkers for monitoring fetal growth status and for the early prediction of fetal overgrowth (Saucedo et al., 2024). Among these, PPARGC1α has received particular attention. This gene is a key regulator of brown and beige adipose tissue formation and function, and its aberrant methylation has been closely linked to fetal adipose tissue development. Evidence from placental tissues of GDM pregnancies indicates that maternal hyperglycemia is associated with altered PPARGC1α methylation, which may impair fetal adipose tissue development and promote excessive lipid accumulation in late gestation, thereby increasing the risk of neonatal obesity (Côté et al., 2016). Importantly, dynamic changes in PPARGC1α DNA methylation detected during early childhood have been shown to predict obesity trajectories during adolescence (Clarke-Harris et al., 2014), suggesting that these early epigenetic alterations are persistent and may represent a key mechanism underlying the elevated long-term metabolic risk observed in infants born LGA to mothers with GDM.

Beyond metabolic phenotypes, early abnormalities in the cardiovascular system have also been observed in LGA offspring of GDM pregnancies. Epidemiological studies have reported that LGA individuals exhibit a significantly elevated risk of developing CVD and metabolic syndrome (MetS) in adulthood (Boney et al., 2005; Evagelidou et al., 2010), although the biological mechanisms remain incompletely understood. Recent clinical evidence has provided novel insights into this association. Cord blood monocytes from neonates born to mothers with GDM display enhanced phagocytic activity and increased adhesive capacity-functional characteristics closely implicated in the pathogenesis of atherosclerosis. Transcriptomic analyses further revealed that CXCL8^+^IL1B^+^ monocytes in cord blood from GDM-exposed neonates exhibit gene expression profiles highly similar to those of myeloid cells within coronary atherosclerotic plaques (Yin et al., 2024), suggesting that intrauterine hyperglycemic exposure may exert long-term effects on offspring cardiovascular health through immune-inflammatory programming. Consistent with these findings, clinical studies have demonstrated early cardiovascular structural remodeling in LGA infants, regardless of maternal GDM status, including increased abdominal aortic intima-media thickness and elevated left ventricular mass. Notably, these abnormalities are not transient but can persist into childhood, as evidenced by sustained carotid intima-media thickness thickening in LGA children aged 8–9 years (Akcakus et al., 2006; Yapicioglu et al., 2023). In parallel, lipid metabolic disturbances present at birth-characterized by elevated TC and low-density lipoprotein cholesterol levels-have been shown to persist through early childhood (3–6 years of age) (Lin et al., 2016). Supporting evidence from animal models further demonstrates that offspring of GDM-exposed dams develop insulin resistance and elevated levels of VLDL in adulthood (Merzouk et al., 2002). Importantly, aberrant DNA methylation of genes involved in cardiovascular function and lipid metabolism has been detected in the cord blood of LGA neonates (Lin et al., 2016), indicating that an adverse molecular foundation for cardiovascular health may already be established early in life.

Taken together, current evidence supports the notion that GDM-related fetal overgrowth and the associated increased risks of cardiovascular disease and metabolic syndrome are closely linked to early-life epigenetic reprogramming. Notably, this process does not appear to be entirely irreversible. Emerging data suggest that modifiable maternal exposures during pregnancy can influence fetal epigenetic states and thereby affect long-term metabolic health in offspring. For instance, maternal exercise interventions have been shown to induce DNA methylation changes in key metabolic regulatory genes, such as POFUT1 and MCEE, in maternal blood, changes that are associated with improved metabolic indicators in neonates (Yan J. et al., 2023). In addition, maternal dietary composition-particularly the intake of methyl-donor nutrients-has been identified as an important and modifiable determinant of fetal epigenetic patterns. Dietary folate and choline intake have been associated with improved insulin sensitivity and a reduced risk of fetal overgrowth, respectively (Bankole et al., 2022).

Overall, epigenetic markers represent a critical and potentially modifiable intermediary linking the intrauterine environment to fetal overgrowth and long-term health outcomes, rather than a fixed genetic endpoint. However, most existing evidence remains associative in nature, and causal relationships between specific epigenetic modifications and long-term metabolic or cardiovascular outcomes have yet to be firmly established. Future studies integrating longitudinal cohort designs, mechanistic experiments, and clinically relevant endpoints will be essential to identify which epigenetic changes are reversible, predictive, and amenable to targeted intervention.

## 5 Prevention and management of GDM-related fetal overgrowth

### 5.1 Early screening and early intervention

Preventive medicine plays a crucial role in safeguarding the future health of populations. Therefore, the most effective strategy for managing GDM-related fetal overgrowth remains “early screening, early intervention, and early treatment”. Early fetal ultrasound monitoring has been shown to help reduce the incidence of adverse pregnancy outcomes (Antoniou et al., 2022). At 34 weeks of gestation, if ultrasound examination reveals a fetal abdominal fat layer (FFL) thickness greater than 0.48 cm in women with GDM, the relative risk of delivering a macrosomic infant increases significantly-more than doubling compared to women with FFL thickness ≤0.48 cm, where the incidence of macrosomia is only 16% (Elessawy et al., 2017). Compared with mothers of non-macrosomic pregnancies, GDM patients with macrosomic pregnancies exhibit significantly increased fetal anterior abdominal wall thickness (FAAWT) between 36 and 39 weeks of gestation. When a cutoff value of FAAWT >6 mm is used for prediction, the sensitivity for detecting macrosomia reaches 87.5%, with a specificity of 75%, a positive predictive value of 40%, and a negative predictive value of 96.9% (Bansal et al., 2023). In 2020, a study first introduced the concept of combining symphysis-fundal height (SFH) and abdominal circumference (AC) to calculate the integrated symphysial fundal height-abdominal circumference (ISFHAC) index as a predictive indicator of pregnancy outcomes. ISFHAC demonstrates significant predictive value for both normal pregnancies and GDM pregnancies complicated by macrosomia. In GDM pregnancies, when the ISFHAC cutoff is set at 41.7, its sensitivity for predicting macrosomia reaches 75.9%, outperforming traditional indicators such as maternal body mass index, SFH or AC measured alone, and gestational age (Chen et al., 2020). Taken together, these ultrasound-derived markers provide clinically meaningful information for early identification of fetuses at increased risk of overgrowth in pregnancies complicated by GDM. Earlier detection of excessive fetal adiposity or accelerated growth trajectories allows clinicians to implement timely and targeted management strategies, including intensified glycemic monitoring, earlier initiation or adjustment of medical nutrition therapy, and, when necessary, initiation of pharmacological intervention. Such ultrasound-guided risk stratification supports a more individualized management approach, enabling clinicians to intervene before excessive fetal growth becomes established, rather than relying solely on late-gestational weight estimates or maternal metabolic indices. However, current evidence supporting the clinical utility of these ultrasound parameters is largely derived from observational studies, and their role in guiding specific therapeutic thresholds or intervention algorithms remains to be fully defined. Although these markers clearly enhance risk assessment and clinical vigilance, further prospective studies are needed to determine how their integration into routine GDM management can most effectively inform treatment intensity and timing, and to what extent such strategies translate into sustained improvements in perinatal outcomes.

GDM screening is typically conducted between weeks 24 and 28 of pregnancy (Yanachkova et al., 2022), but in pregnant women diagnosed with GDM, approximately 30%–70% may exhibit abnormal blood glucose elevation as early as before week 20 of pregnancy (Sweeting et al., 2024), suggesting that endocrine dysfunction may already be present in both the mother and fetus during early pregnancy. Although the typical clinical feature of GDM is glucose metabolism abnormalities that appear in the mid-to-late stages of pregnancy, some scholars have pointed out that before the clinical diagnosis of GDM is established, placental development may already be affected by abnormally elevated levels of insulin, IGF, and leptin (Qiu et al., 2005; D’Anna et al., 2007). Research indicates that weeks 12–14 of pregnancy may be a critical window period for increased placental sensitivity to maternal insulin, primarily due to high insulin receptor expression in STB cells during early pregnancy, which significantly decreases by term. In early pregnancy, maternal insulin secretion is closely associated with placental weight, which in turn is highly correlated with neonatal birth weight (O’Tierney-Ginn et al., 2015; Sathasivam et al., 2023). Research data indicate that compared to women diagnosed with GDM between 22 and 30 weeks of gestation, those diagnosed after 30 weeks have a significantly increased risk of delivering LGA infants (Regnault et al., 2024). Therefore, compared to interventions implemented in the late stages of pregnancy, initiating intervention protocols in the early stages of pregnancy can effectively reduce the incidence of neonatal hyperlipidemia, macrosomia, LGA, and SGA (Calancie et al., 2025). Overall, these findings suggest that metabolic dysregulation in GDM begins earlier than its conventional clinical diagnosis, with early pregnancy representing a critical window during which placental insulin sensitivity and endocrine signaling may already shape fetal growth trajectories. This temporal mismatch between early pathophysiology and mid-gestation screening underscores the potential value of earlier risk stratification and timely intervention to mitigate excessive fetal growth and adverse neonatal metabolic outcomes.

### 5.2 Integrated management through diet and exercise

GDM-related fetal overgrowth is generally considered to occur within the broader context of impaired maternal metabolic homeostasis during pregnancy, rather than arising from isolated abnormalities in fetal growth signaling. Based on this understanding, clinical and translational studies increasingly recognize that interventions targeting GDM-related fetal overgrowth should not focus solely on glycemic control, but instead aim to improve the overall maternal metabolic environment during pregnancy. Accordingly, recent research has placed greater emphasis on modifiable maternal factors-particularly dietary patterns and lifestyle behaviors-as potential intervention targets for reducing the risk of fetal overgrowth in GDM pregnancies.

In recent years, an integrated management model known as the 1-day care clinic has been widely established and implemented worldwide (Wang et al., 2022). Unlike conventional management strategies that primarily focus on glycemic monitoring and individualized follow-up, the 1-day care clinic integrates structured nutritional education, standardized exercise guidance, and scenario-based practical training to enhance pregnant women’s understanding of dietary composition, energy distribution, and appropriate physical activity, while also improving their awareness of GDM-related maternal and neonatal risks. This comprehensive approach facilitates more effective adherence to medical advice and sustained self-management, thereby contributing to improved gestational weight control and mitigation of metabolic disturbances. Clinical studies have demonstrated that this integrated management model not only reduces fasting blood glucose, postprandial glucose, and HbA1c levels, but, importantly, is also associated with a significant reduction in the incidence of macrosomia. These findings suggest that behavior-centered, structured management strategies are both feasible and clinically valuable for reducing the risk of GDM-related fetal overgrowth (Cao et al., 2021; Wang et al., 2022; Xu et al., 2025). Recent studies have shown that the DASH diet has demonstrated significant benefits for weight control and body fat improvement, making it a potential nutritional intervention strategy for GDM. It emphasizes a diet rich in fruits, vegetables, and low-fat dairy products while reducing the intake of saturated fats and total fats (Soltani et al., 2016). In a recent meta-analysis, 2, 712 pregnant women participated in a randomized controlled trial, indicating that the DASH diet significantly reduced fasting blood glucose and 2hPG levels. In terms of reducing the incidence of macrosomia, the DASH diet not only outperformed the control group but also outperformed the high-fiber diet, antioxidant-enriched diet, and structured exercise groups (Zhang L. et al., 2024). These findings indicate that the DASH diet represents a highly promising intervention for improving pregnancy outcomes in women with GDM. Such evidence provides practical support for the feasibility of reducing the risk of GDM-related fetal overgrowth through dietary modification and behavioral interventions that improve the maternal metabolic environment. However, it is important to note that current evidence mainly originates from observational studies or integrated intervention trials, making it difficult to disentangle the independent effects of individual intervention components. Moreover, the specific biological mechanisms by which these interventions influence placental function and fetal growth regulation remain to be further elucidated.

Gestational insulin resistance primarily occurs at the skeletal muscle level, and prenatal exercise interventions-particularly those engaging large muscle groups-can enhance maternal glucose metabolism and improve insulin sensitivity. These physiological benefits contribute to the prevention of GDM, as well as prenatal anxiety and depression, and excessive gestational weight gain (Mottola and Artal, 2016; Ribeiro et al., 2022). Numerous studies have demonstrated that regular physical activity provides clear clinical benefits for women with GDM. The currently recommended exercise regimens for GDM patients mainly include two types: aerobic exercise and resistance training (Sklempe Kokic et al., 2018; Jin et al., 2022). Research has shown that prenatal exercise interventions can significantly reduce placental expression levels of GLUT-1 in GDM patients, thereby effectively mitigating the adverse developmental effects of GDM on the offspring’s heart, liver, and kidneys (Tang et al., 2024). Animal studies have further confirmed that exercise during pregnancy can ameliorate metabolic disturbances in GDM mice, increase pancreatic β-cell mass, and help maintain intestinal microbiota homeostasis (Mahizir et al., 2020). Exercise interventions have also been found to significantly lower the risk of macrosomia in offspring of GDM mothers. Compared with diet management alone, a combination of exercise and dietary intervention is more effective in reducing the incidence of macrosomia among GDM pregnancies (Wang et al., 2015). Importantly, while prenatal exercise effectively reduces the risk of LGA and macrosomia, it does not elevate the risk of SGA births. Moreover, maternal exercise during pregnancy contributes to long-term protection against chronic metabolic diseases in offspring (Vargas-Terrones et al., 2019). Therefore, regular exercise, as a fundamental intervention in GDM management, holds significant clinical value in improving both maternal and neonatal outcomes. However, many pregnant women still struggle to maintain consistent exercise routines. Thus, clinical practice should prioritize education and promotion efforts to encourage adherence to prenatal exercise interventions. Current evidence supports prenatal physical activity as an important lifestyle intervention for reducing the risk of fetal overgrowth in pregnancies complicated by GDM. Regular exercise may improve maternal insulin sensitivity and overall metabolic status, thereby indirectly modulating the intrauterine nutritional environment and associating with lower rates of macrosomia and LGA. Compared with dietary intervention alone, combined exercise and dietary management appears to confer more consistent benefits in limiting excessive fetal growth. However, the implementation of exercise interventions in clinical practice remains challenged by suboptimal adherence and heterogeneity in exercise protocols. The optimal type, intensity, and duration of exercise, as well as their differential effects across maternal metabolic backgrounds, require further clarification through larger and more standardized studies.

### 5.3 Future therapeutic perspectives and translational implications

Current clinical screening and management of GDM are primarily guided by international recommendations issued by the International Association of Diabetes and Pregnancy Study Groups (IADPSG), the American Diabetes Association (ADA), and the National Institute for Health and Care Excellence (NICE). These guidelines advocate a stepwise, glucose-centered management paradigm, beginning with lifestyle modification and escalating to pharmacological therapy when glycemic targets are not achieved (Metzger et al., 2008; Metzger et al., 2010; National Institute for Health and Care Excellence, 2018; American Diabetes Association Professional Practice Committee, 2024). While such approaches effectively reduce maternal hyperglycemia, growing clinical evidence indicates that fetal overgrowth-manifesting as macrosomia or LGA-remains common even in pregnancies with apparently well-controlled maternal glucose levels (Mackin et al., 2018; He et al., 2023). This observation underscores a critical limitation of glucose-focused management and suggests that mechanisms beyond maternal glycemia, particularly placental metabolic dysregulation and vascular dysfunction, play a decisive role in driving excessive maternofetal nutrient transfer and abnormal fetal growth.

Within current guidelines, insulin is regarded as the first-line pharmacological therapy for GDM due to its lack of placental transfer. However, barriers related to injection burden, cost, and treatment adherence have prompted continued interest in oral antihyperglycemic agents. Among these, metformin has attracted particular attention not merely for its glucose-lowering efficacy, but for its reproducible association with reduced rates of fetal overgrowth and neonatal hypoglycemia in GDM pregnancies (Salomäki et al., 2013; Alfadhli, 2015). Importantly, these clinical benefits often appear disproportionate to improvements in maternal glycemic indices, implying that metformin may exert additional effects through direct modulation of placental function and maternofetal nutrient transport. Nevertheless, because metformin readily crosses the placenta, concerns regarding potential long-term metabolic programming effects in offspring remain unresolved, highlighting the need for careful benefit-risk assessment and patient stratification when prevention of fetal overgrowth is the primary therapeutic objective.

In this context, emerging therapeutic frameworks are increasingly shifting from exclusive normalization of maternal glucose levels toward mechanism-based interventions that directly target placental dysfunction as a strategy to restrain excessive fetal growth. Beyond its systemic metabolic effects, metformin activates AMPK and suppresses placental mTOR signaling, resulting in downregulation of glucose and amino acid transporters, attenuation of placental inflammation, and limitation of excessive nutrient flux to the fetus-mechanistic actions that closely align with its clinically observed reduction in macrosomia risk (Pérez-Pérez et al., 2008; Hung et al., 2021). Concurrently, placental vascular dysfunction and chronic inflammation represent additional, glucose-independent drivers of fetal overgrowth. Hydrophilic statins with low placental permeability, such as pravastatin, have been shown to improve placental endothelial function, restore angiogenic balance, and dampen inflammatory signaling, thereby offering a potential vascular-targeted approach to modulating abnormal fetal growth (Costantine et al., 2016; Lefkou et al., 2016; Ahmed et al., 2020), although direct clinical evidence in GDM remains limited.

Future therapeutic frameworks should shift toward stratified, mechanism-based precision intervention. For patients who already exhibit placental metabolic hyperactivity or abnormal activation of the mTOR pathway, adjunctive use of placental-modulating agents such as metformin may be considered after comprehensive risk assessment. For patients presenting predominantly with vascular endothelial dysfunction and chronic inflammation, low-dose pravastatin and other vascular-targeted drugs may hold greater therapeutic potential. The successful implementation of such strategies requires establishing a biomarker system for early detection of placental dysfunction, including ultrasound Doppler assessment of placental blood flow and analysis of placenta-specific metabolites via omics technologies, as well as conducting prospective clinical trials with placental function as a core endpoint. Ultimately, building an integrated management model that combines glycemic control, targeted placental modulation, and fetal growth monitoring is expected to more effectively prevent GDM-related fetal overgrowth and may positively influence the long-term health of offspring by improving the intrauterine metabolic environment. Recent and ongoing clinical trials evaluating such stratified approaches are summarized in Table 2.

**TABLE 2:** Registered clinical trials related to GDM and macrosomia (last 5 years).

Scientific title	Study type/Model	Study start	Target sample size	Interventions	Outcome evaluation method	Registration number	Additional information
Preventive effect of probiotics in GDM	Interventional (Randomized clinical trial)	2023-10-27	334	Intervention group: 1 bag of probiotics powder per day Control group: Placebo 1 bag per day	Primary outcome measures: Perform an OGTT at weeks 24–28 of gestation and record perinatal results regarding newborn weight Secondary outcome measures: The incidence of macrosomia	NCT06938464	Gestational time points: Intervention from 12–16 weeks until OGTT at 24–28 weeks Outcome definition (Macrosomia): Newborn weight >4 kg Status: Ongoing
Influence of triglyceride-glucose (TyG) index and TG/HDL-C ratio on fetal macrosomia	Observational Model (Case-Control)	2023-01-01	302	Blood samples were collected from pregnant women in the study and control group between the 28th and 40th weeks of pregnancy	Primary outcome measures: The TyG index; The triglyceride to high density lipoprotein cholesterol ratio Secondary outcome measures: HOMA-IR was calculated according to the formula: fasting insulin (microU/L) x fasting glucose (nmol/L)/22.5	NCT06463990	Gestational time points: Blood collection: 28–40 weeks of pregnancy Outcome definition (Macrosomia): Per study protocol.: Not specified Status: Completion
Timing of ambulation and infant birth weight in gestational diabetes	Interventional (Randomized clinical trial)	2022-10-01	90	Experimental group: Participants will be counseled to either complete 20 min of walking after meals No Intervention group: Participants will be counseled with routine exercise counseling of 30 min of low-impact 5x a week	Primary outcome measures: Infant birthweight percentile Secondary outcome measures: infant birthweight, mode of delivery, fetal macrosomia, neonatal hypoglycemia treatment	NCT06157684	Gestational time points: Intervention after GDM diagnosis until delivery Outcome definition (Macrosomia): Per study protocol Status: Completion
The efficacy of fetal arterial and venous doppler indices in predicting perinatal outcome	Observational Model (Case-Crossover)	2022-06-15	140	Diagnostic Test: Fasting blood sugar, 2 h postprandial, HbA1c, Ultrasound and Doppler study	Primary outcome measures: The diagnostic value of umbilical artery (UA) Doppler indices and middle cerebral artery (MCA) Doppler indices in predicting adverse perinatal outcome among diabetic pregnant patients Secondary outcome measures: The diagnostic value of cerebroplacental ratio (CPR) and ductus venosus Doppler indices in predicting adverse perinatal outcome among diabetic pregnant patients	NCT05410080	Gestational time points: Doppler study within 24 h before pregnancy termination (34–39 weeks) Outcome definition (Macrosomia): Birth weight >90th percentile for gestational age Status: Completion
The study of biomarker in early diagnosis of GDM by metabolomics	Observational Model (Cohort)	2022-05-04	100	All subjects had a 5 mL blood sample collected from each subject at each of four time points during pregnancy (6–13 weeks, 24–28 weeks, 36 weeks prior to delivery, and 6 weeks post-delivery)	Primary outcome measures: The OGTT test (75 g glucose) was performed between 24 and 28 weeks of gestation	NCT05733195	Gestational time points: Blood collection: 6–13 weeks, 24–28 weeks, 36 weeks (pre-delivery), 6 weeks (postpartum) Outcome definition (Macrosomia): Listed as a GDM-related risk Status: Completion
Strict versus permissive thresholds for initiation of pharmacotherapy in gestational diabetes (START 2)	Interventional (Randomized clinical trial)	2024-09-01	430	Drug: Insulin; Strict threshold (two abnormal values or more over a 1-week period) vs. Permissive threshold (50% of values elevated over 1 week)	Primary outcome measures: Neonatal composite, including: LGA of neonate defined as birth weight >90th percentile for gestational age using the Fenton growth chart, hypoglycemia, hyperbilirubinemia, stillbirth or neonatal death, birth trauma Secondary outcome measures: Gestational Age of Birth, APGAR Score, Brachial Plexus Injury, Respiratory distress, Maternal hypoglycemia, Shoulder Dystocia, Obstetric anal sphincter injury (OASIS), Cesarean Delivery, Postpartum hemorrhage, etc.	NCT06419803	Gestational time points: Intervention after GDM diagnosis until delivery Outcome definition (Macrosomia/LGA): Birth weight >90th percentile for gestational age Status: Ongoing
D-chiro inositol in prevention of gestational diabetes mellitus in China	Interventional (Randomized clinical trial)	2021-01-01	291	Active comparator: D-chiro inositol 500 mg twice a day Placebo comparator: Placebo (similar appearance but not containing myo-inositol) 500 mg twice a day	Primary outcome measures: The number of cases and incidence of GDM according to OGTT. Secondary outcome measures: The incidence of macrosomia (macrosomia refers to a newborn weigh more than 4 kg), weight gain during pregnancy, the Caesarean-section incidence	NCT04801485	Gestational time points: Intervention from 12–16 weeks until OGTT at 24–28 weeks Outcome definition (Macrosomia): Newborn weighs more than 4 kg Status: Completion

## 6 Summary and outlook

As discussed above, the pathogenesis of GDM-related fetal overgrowth involves multiple interconnected pathophysiological mechanisms. Current research has identified several key components of this process, including enhanced placental nutrient transport and aberrant activation of fetal growth-related signaling pathways, such as the IGF and mTOR pathways. In addition to these classical mechanisms, accumulating evidence indicates that pathological changes-such as abnormal placental angiogenesis, and imbalances in trophoblast cell proliferation and apoptosis-also contribute to fetal overgrowth. Recent advances have further expanded the traditional understanding of this condition, confirming that gut microbiota dysbiosis and EVs-mediated intercellular communication also play significant roles. However, important scientific questions such as how these newly discovered factors interact with classical mechanisms and whether cascade amplification effects exist across systems remain to be further explored. Additionally, epigenetic studies provide new perspectives for understanding the long-term health impacts of macrosomia, suggesting that intrauterine hyperglycemia exposure may be associated with an increased risk of CVD and MetS in adulthood (Lin et al., 2016; Taschereau et al., 2023). Recent studies also indicate that intrauterine hyperglycemia leads to reduced fetal fertility and affects fetal sexual differentiation through epigenetic reprogramming (Cong et al., 2025). However, there is currently no definitive research demonstrating whether GDM-related fetal overgrowth exhibits gender-specific differences or a decline in reproductive capacity. Due to the need for long-term follow-up observations in such studies, the available evidence remains limited. On the other hand, animal models of GDM have relatively homogeneous genetic backgrounds, whereas humans possess highly diverse genetic profiles. It is challenging to replicate the complex genetic background and gene-environment interactions of humans in animal models, which limits the translation of research findings into clinical practice. Nevertheless, in-depth exploration of the transgenerational epigenetic effects of GDM on offspring health holds profound scientific value and clinical significance.

In clinical practice, early screening and dynamic monitoring of GDM are emphasized as essential strategies. Ultrasonic indicators (such as FFL thickness and ISFHAC) provide crucial evidence for assessing fetal growth trends. Regarding interventions, lifestyle modifications (such as the DASH diet and physical activity during pregnancy) have demonstrated potential in reducing the risk of fetal overgrowth by regulating glucose and lipid metabolism and improving placental function. In the field of pharmacotherapy, metformin has shown promise due to its unique role in enhancing insulin sensitivity and its clinical evidence supporting reductions in the risks of macrosomia and LGA infants. As such, metformin represents a viable alternative to insulin therapy (Guo et al., 2019; Kirovakov et al., 2024). Furthermore, mechanism-based intervention strategies offer promising avenues for advancing precision medicine in the management of GDM, although their safety and efficacy require further research validation before widespread clinical application.

However, the clinical management of GDM still faces significant challenges. Although blood glucose-based interventions have led to notable improvements in maternal metabolic parameters, the incidence of fetal overgrowth remains high, highlighting the limitations of current treatment strategies. This situation arises primarily from two interrelated issues. First, many studies have overly focused on blood glucose control as a singular factor, neglecting the fact that GDM-related fetal overgrowth is a multifactorial pathological process. Contributing factors include maternal pre-pregnancy overweight or obesity, advanced maternal age, and mental health issues during pregnancy (OuYang et al., 2021; Song et al., 2022; Zhang T. et al., 2024)-all of which significantly elevate the risk of fetal overgrowth. Second, fetal overgrowth remains under-prioritized as an adverse clinical outcome in routine clinical practice. To address these challenges, future research must transition from a unidimensional focus on glycemic control to a more comprehensive, integrated approach that considers multiple dimensions, including maternal metabolic status, placental function regulation, and fetal growth signaling pathways. Such a multidisciplinary strategy holds the potential to enable the development of more targeted and effective interventions, ultimately achieving improved outcomes for both mothers and their fetuses.
